# Supplementary material for: Examining area-level variation in service organisation and delivery across the breadth of primary healthcare. Usefulness of measures constructed from routine data
Source: PLoS One. 2021 Dec 1;16(12):e0260615. doi: 10.1371/journal.pone.0260615 (PMC8635352; doi:10.1371/journal.pone.0260615)
Supplement: S1 Appendix — (DOCX) [file pone.0260615.s001.docx]

**ONLINE SUPPORTING SUPPLEMENTARY FILES**

Table S1. Non-referred GP attendance MBS item numbers included in the analysis

| **Item Group Description** | **Item Numbers** |
| --- | --- |
| Brief consultations | 3 and 52 |
| Standard consultations | 23 and 53 |
| Long consultations | 36 and 54 |
| Prolonged consultation | 44 and 57 |
| Remainder A1 and A2 | 4, 20, 24, 35, 37, 43, 47, 51,58–60, 65, 92, 93, 95, 96 |
| After-hours services (urgent and non- urgent) | 597, 598, 559, 600, 5000, 5003, 5010, 5020, 5023, 5028, 5040, 5043, 5049, 5060, 5063, 5067, 5200, 5203, 5207, 5208, 5220, 5223, 5227, 5228, 5260, 5263, 5265, 5267 |
| Heath assessments | 701, 703, 705, 707, 715 |
| GP management plans | 721, 723, 729, 731, 732 |
| Cervical screening (for unscreened or significantly underscreened women) | 2497, 2501, 2503, 2504, 2506, 2507, 2509, 2598, 2600, 2603, 2606, 2610, 2613, 2616 |
| Asthma cycle of care | 2546, 2547, 2552, 2553, 2558, 2559, 2664, 2666, 2668,2673, 2675, 2677 |
| Diabetes cycle of care | 2517, 2518, 2521, 2522, 2525, 2526, 2620, 2622, 2624, 2631, 2633, 2635 |

Note: Remainder A1 and A2 refers to non-referred general practice services provided by vocationally and non-vocationally registered medical practitioners at a residential care facility or at other than consulting rooms or residential aged care facility.

Table S2. Correlation between measures within dimensions—availability, affordability and comprehensiveness/coordination

| **Availability** | ***AMPCo Count*** | | ***AMPCo FTE*** | | ***AIHW FTE*** | |
| --- | --- | --- | --- | --- | --- | --- |
|  | **r** | **p** | **r** | **p** | **r** | **P** |
| *AMPCo FTE* | 1.00 | <.001 | – | – | – | – |
| *AIHW FTE* | 0.33 | 0.002 | 0.30 | 0.004 | ­ | – |
| *PHIDU FWE* | -0.07 | 0.529 | -0.03 | 0.755 | 0.07 | 0.781 |
| **Affordability** | ***OOP*** | | ***Bulk-billing*** | | ***HCC*** | |
|  | **r** | **p** | **r** | **p** | **r** | **P** |
| *Bulk-billing* | -0.93 | <.001 | – | – | – | – |
| *HCC holders* | -0.35 | <.001 | 0.31 | 0.004 | – | – |
| *PC holders* | -0.21 | 0.044 | 0.14 | 0.187 | 0.87 | <.001 |
| **Comprehensiveness/ Coordination** | ***CD care*** | | ***Health Assessments*** | | ***Cervical screening*** | |
|  | **r** | **p** | **r** | **p** | **r** | **p** |
| *Health assessments* | 0.56 | <.001 | – | – | – | – |
| *Cervical screening* | -0.28 | 0.008 | -0.09 | 0.376 | – | – |
| *CHC* | -0.17 | 0.127 | 0.04 | 0.695 | -0.09 | 0.437 |

Abbrev: SD, standard deviation; PHC, primary health care; SE, socioeconomic; AMPCo; Australian Medical Publishing Company; AIHW, Australian Institute of Health and Welfare; PHIDU, Public Health Information Development Unit; FTE, full-time equivalent; FWE, full-time workload equivalent; OOP, out-of-pocket expenses; CD, chronic disease; HA, health assessment; CHC, community health centre; r, Pearson’s correlation coefficient; p, p‑value for coefficient. FTE and FWE per 1000 usual resident population.

Table S3. Correlation between availability measures separately by region

| **Region** | ***AMPCo count*** | | ***AMPCo FTE*** | | ***AIHW FTE*** | |
| --- | --- | --- | --- | --- | --- | --- |
|  | **r** | **p** | **r** | **p** | **r** | **p** |
| **Major Cities** |  |  |  |  |  |  |
| *AMPCo FTE* | 1.00 | <.001 | – | – | – | – |
| *AIHW FTE* | 0.49 | <.001 | 0.50 | <.001 | ­ | – |
| *PHIDU FWE* | -0.69 | <.001 | -0.66 | <.001 | -0.49 | <.001 |
| **Inner regional** |  |  |  |  |  |  |
| *AMPCo FTE* | 1.00 | <.001 | – | – | – | – |
| *AIHW FTE* | 0.62 | <.001 | 0.63 | <.001 | – | – |
| *PHIDU FWE* | 0.23 | 0.221 | 0.26 | 0.181 | 0.38 | 0.050 |
| **Outer regional/remote** |  |  |  |  |  |  |
| *AMPCo FTE* | 1.00 | <.001 | – | – | – | – |
| *AIHW FTE* | -0.34 | 0.113 | -0.36 | 0.092 | – | – |
| *PHIDU FWE* | 0.43 | 0.040 | 0.45 | 0.030 | -0.14 | 0.535 |

Abbrev. AMPCo; Australian Medical Publishing Company; AIHW, Australian Institute of Health and Welfare; PHIDU, Public Health Information Development Unit; FTE, full-time equivalent; FWE, full-time workload equivalent; r, Pearson’s correlation coefficient; p, p-value. FTE and FWE per 1000 usual resident population.

Table S4. Area PHC service characteristic quartiles: SA3 composition and mean (SD) and median (IQR) values

| **PHC service characteristic by quartile** | **Definition** | **SA3 composition** | | **Values** | |
| --- | --- | --- | --- | --- | --- |
|  |  | **No. SA3s** | **% all SA3s** | **Mean (SD)** | **Median (IQR)** |
| ***AIHW FTE*** | FTE GPs/1000 URP |  |  |  |  |
| 1st quartile |  | 31 | 34.1 | 0.6 (0.2) | – |
| 2nd quartile |  | 20 | 22.0 | 0.8 (0.0) | – |
| 3rd quartile |  | 19 | 20.9 | 0.9 (0.0) | – |
| 4th quartile |  | 21 | 23.1 | 1.1 (0.2) | – |
| Total |  | 91 | 100 | 0.8 (0.2) | 0.9 (0.3) |
| ***OOP expenses*** | OOP costs/service (AUD) |  |  |  |  |
| 1st quartile |  | 17 | 18.7 | 0.3 (0.3) | – |
| 2nd quartile |  | 28 | 30.8 | 2.3 (0.8) | – |
| 3rd quartile |  | 22 | 24.2 | 4.9 (0.7) | – |
| 4th quartile |  | 23 | 25.3 | 8.6 (2.9) | – |
| Missing |  | 1 | 1.1 | – | – |
| Total (non-missing) |  | 90 | 100 | 4.2 (3.4) | 3.7 (4.9) |
| ***Bulk-billing*** | % all GP services bulk-billed |  |  |  |  |
| 1st quartile |  | 24 | 26.4 | 69.5 (5.9) | – |
| 2nd quartile |  | 21 | 23.1 | 80.6 (2.0) | – |
| 3rd quartile |  | 27 | 29.7 | 89.0 (2.7) | – |
| 4th quartile |  | 16 | 17.6 | 97.0 (1.5) | – |
| Missing |  | 3 | 3.3 | – | – |
| Total (non-missing) |  | 90 | 100 | 83.1 (10.6) | 83.2 (15) |
|  |  |  |  |  |  |
| ***After-hours care*** | Percentage of all GP services claimed after hours |  |  |  |  |
| 1st quartile |  | 31 | 34.1 | 0.7 (0.5) | – |
| 2nd quartile |  | 22 | 24.2 | 3.5 (1.0) | – |
| 3rd quartile |  | 18 | 19.8 | 6.8 (0.8) | – |
| 4th quartile |  | 19 | 20.9 | 9.8 (1.8) | – |
| Missing |  | 1 | 1.1 | – | – |
| Total (non-missing) |  | 90 | 100 | 4.4 (3.6) | 3.7 (6.5) |
| ***Health assessments*** | No. health assessments claimed/100 eligible population^1^ |  |  |  |  |
| 1st quartile |  | 24 | 26.4 | 5.5 (1.8) | – |
| 2nd quartile |  | 19 | 20.9 | 7.8 (0.4) | – |
| 3rd quartile |  | 22 | 24.2 | 9.7 (0.8) | – |
| 4th quartile |  | 25 | 27.5 | 13.8 (2.6) | – |
| Missing |  | 1 | 1.1 | – | – |
| Total (non-missing) |  | 90 | 100 | 9.3 (3.6) | 6.7 (4.3) |
|  |  |  |  |  |  |
|  |  |  |  |  |  |
|  |  |  |  |  |  |
|  |  |  |  |  |  |
|  |  |  |  |  |  |
| ***Cervical screening*** | % of women aged 20–69 who received a Pap smear in the last 2 years |  |  |  |  |
| 1st quartile |  | 20 | 22.0 | 49.6 (2.1) | – |
| 2nd quartile |  | 23 | 25.3 | 53.9 (1.0) | – |
| 3rd quartile |  | 25 | 27.5 | 57.6 (0.8) | – |
| 4th quartile |  | 23 | 25.3 | 63.6 (4.3) | – |
| Total |  | 91 | 100 | 56.4 (5.6) | 56.1 (6.8) |
| ***CD care*** | Number of CD items claimed/100 eligible population^2^ |  |  |  |  |
| 1st quartile |  | 26 | 21.98 | 11.5 (3.9) | – |
| 2nd quartile |  | 23 | 25.27 | 20.0 (1.9) | – |
| 3rd quartile |  | 19 | 27.47 | 26.8 (1.6) | – |
| 4th quartile |  | 21 | 25.27 | 34.1 (5.8) | – |
| Missing |  | 2 | 1.2 | – | – |
| Total (non-missing) |  | 89 | 100 | 22.3 (9.3) | 21.9 (13.5) |
|  |  |  |  |  |  |
| ***Community Health Centres*** | No. CHC/100,000 URP |  |  |  |  |
| 1st quartile |  | 17 | 18.7 | 2.7 (0.8) | – |
| 2nd quartile |  | 20 | 22 | 4.9 (0.6) | – |
| 3rd quartile |  | 21 | 23.1 | 7.2 (0.9) | – |
| 4th quartile |  | 27 | 29.7 | 14.3 (5.4) | – |
| Missing |  | 6 | 6.6 | – | – |
| Total |  | 85 | 100 | 8.0 (5.5) | 6.4 (5.1) |

Abbrev. HC, primary health care; SA3, statistical area 3; SD, standard deviation; IQR, interquartile range; No., number; %, percentage; yo, years old; OOP, out-of-pocket; HCC, health care card; CD, chronic disease; HC, health centre. ^1^Persons aged 3–5 years, 45–49 years, 75 years and over, Aboriginal and Torres Strait Islander peoples. ^2^Persons reporting a long-term condition. All categories population weighted using 2006 usual resident population (URP).

Table S5. Correlation between area PHC service characteristic measures from each dimension (and components of access) by region

| **Major cities** |  |  |  |  |  |  |  |  |  |
| --- | --- | --- | --- | --- | --- | --- | --- | --- | --- |
| **Dimension** | **PHC characteristic** | **Availability** | | **Affordability** | | | | **Accommodation** | |
|  |  | *GP FTE per capita* | | *OOP expenses* | | *Bulk-billing* | | *After-hours care* | |
|  |  | **r** | **p** | **r** | **p** | **r** | **p** | **r** | **p** |
| **Affordability** | *OOP expenses* | 0.45 | 0.004 | – | – | – | – | – | – |
|  | *Bulk-billing* | -0.41 | 0.008 | – | – | – | – | – | – |
| **Accommodation** | *After-hours care* | -0.28 | 0.085 | -0.51 | <.001 | 0.49 | <.001 | – | – |
| **Comprehensive- ness/ coordination** | *Health assessments* | 0.07 | 0.658 | -0.15 | 0.368 | 0.03 | 0.832 | 0.00 | 0.993 |
|  | *Cervical screening* | 0.30 | 0.057 | 0.80 | <.001 | -0.90 | <.001 | -0.42 | 0.007 |
|  | *CD care* | 0.01 | 0.963 | -0.66 | <.001 | 0.69 | <.001 | 0.40 | 0.011 |
|  | *CHC* | -0.05 | 0.487 | 0.11 | 0.487 | -0.13 | 0.42 | -0.07 | 0.675 |
| **Inner regional** |  |  |  |  |  |  |  |  |  |
| **Dimension** | **PHC characteristic** | **Availability** | | **Affordability** | | | | **Accommodation** | |
|  |  | *GP FTE per capita* | | *OOP expenses* | | *Bulk-billing* | | *After-hours care* | |
|  |  | **r** | **p** | **r** | **p** | **r** | **p** | **r** | **p** |
| **Affordability** | *OOP expenses* | -0.29 | 0.140 | – | – | – | – | – | – |
|  | *bulk-billing* | 0.33 | 0.100 | – | – | – | – | – | – |
| **Accommodation** | *After-hours care* | 0.14 | 0.501 | -0.31 | 0.115 | 0.34 | 0.089 | – | – |
| **Comprehensive- ness/ coordination** | *Health assessments* | 0.21 | 0.284 | 0.15 | 0.458 | 0.03 | 0.903 | -0.20 | 0.310 |
|  | *Cervical screening* | 0.17 | 0.378 | 0.11 | 0.601 | -0.07 | 0.740 | 0.27 | 0.197 |
|  | *CD care* | 0.01 | 0.963 | -0.66 | <.001 | 0.69 | <.001 | 0.40 | 0.011 |
|  | *CHC* | -0.06 | 0.782 | -0.25 | 0.236 | 0.26 | 0.211 | 0.06 | 0.793 |
| **Outer regional/ remote** | |  |  |  |  |  |  |  |  |
| **Dimension** | **PHC characteristic** | **Availability** | | **Affordability** | | | | **Accommodation** | |
|  |  | *GP FTE per capita* | | *OOP expenses* | | *Bulk-billing* | | *After-hours care* | |
|  |  | **r** | **p** | **r** | **p** | **r** | **p** | **r** | **p** |
| **Affordability** | *OOP expenses* | 0.34 | 0.109 | – | – | – | – | – | – |
|  | *Bulk-billing* | -0.28 | 0.203 | – | – | – | – | – | – |
| **Accommodation** | *After-hours care* | -0.35 | 0.097 | -0.20 | 0.350 | 0.38 | 0.082 | – | – |
| **Comprehensive- ness/ coordination** | *Health assessments* | 0.05 | 0.818 | -0.52 | 0.013 | 0.73 | <.001 | 0.11 | 0.608 |
|  | *Cervical screening* | -0.25 | 0.252 | 0.15 | 0.500 | -0.52 | 0.014 | 0.33 | 0.123 |
|  | *CD care* | -0.08 | 0.734 | -0.41 | 0.057 | 0.58 | 0.004 | 0.24 | 0.283 |
|  | *CHC* | -0.14 | 0.536 | -0.06 | 0.515 | -0.15 | 0.515 | -0.39 | 0.077 |

Abbrev. GP, general practitioner; PHC, primary health care; SE, socioeconomic; OLS, ordinary least squares; FTE, full-time equivalent; OOP, out-of-pocket expenses; CD, chronic disease; HA, health assessment; CHC, community health centre; r, Pearson’s correlation coefficients; p, p-value. Per capita: per 1000 usual resident population.
